# Predictors of locating women six to eight years after contact: internet resources at recruitment may help to improve response rates in longitudinal research

**DOI:** 10.1186/1471-2288-7-22

**Published:** 2007-06-18

**Authors:** Suzanne M Cadarette, Leigh Dickson, Monique AM Gignac, Dorcas E Beaton, Susan B Jaglal, Gillian A Hawker

**Affiliations:** 1 Osteoporosis Research Program, Women's College Hospital, Toronto, Canada; 2 Department of Health Policy, Management and Evaluation, University of Toronto, Toronto, Canada; 3 Health Care and Outcomes Research, University Health Network, Toronto, Canada; 4 Department of Public Health Sciences, University of Toronto, Toronto, Canada; 5 Institute for Work and Health, Toronto, Canada; 6 Mobility Program Clinical Research Unit, St. Michael's Hospital, Toronto, Canada; 7 Department of Physical Therapy, University of Toronto, Toronto, Canada; 8 Division of Rheumatology, Women's College Hospital, Toronto, Canada

## Abstract

**Background:**

The ability to locate those sampled has important implications for response rates and thus the success of survey research. The purpose of this study was to examine predictors of locating women requiring tracing using publicly available methods (primarily Internet searches), and to determine the additional benefit of vital statistics linkages.

**Methods:**

Random samples of women aged 65–89 years residing in two regions of Ontario, Canada were selected from a list of those who completed a questionnaire between 1995 and 1997 (n = 1,500). A random sample of 507 of these women had been searched on the Internet as part of a feasibility pilot in 2001. All 1,500 women sampled were mailed a newsletter and information letter prior to recruitment by telephone in 2003 and 2004. Those with returned mail or incorrect telephone number(s) required tracing. Predictors of locating women were examined using logistic regression.

**Results:**

Tracing was required for 372 (25%) of the women sampled, and of these, 181 (49%) were located. Predictors of locating women were: younger age, residing in less densely populated areas, having had a web-search completed in 2001, and listed name identified on the Internet prior to recruitment in 2003. Although vital statistics linkages to death records subsequently identified 41 subjects, these data were incomplete.

**Conclusion:**

Prospective studies may benefit from using Internet resources at recruitment to determine the listed names for telephone numbers thereby facilitating follow-up tracing and improving response rates. Although vital statistics linkages may help to identify deceased individuals, these may be best suited for post hoc response rate adjustment.

## Background

Participation in epidemiologic studies has been declining [1]. Ability to locate those sampled for a new study or to trace participants for longitudinal follow-up has important implications for response rates and thus the success of survey research. Prior evidence suggests that response rates among those who move are similar to those who do not, provided the "movers" are successfully located [2]. It is recommended that participants in longitudinal studies be contacted every six to twelve months to maintain current address information [2,3]. The feasibility of follow-up among those not contacted for longer periods becomes questionable if a standardized source, such as a clinical or administrative database, with updated address information, is not available [4,5]. This may be particularly true when tracing women, as prior research has found that fewer than one third of known addresses for women were identified using searches of their name on the Internet [6]. However, 82% were identified using reverse lookup strategies (i.e., searching their telephone number or address); 41% were listed under a male's name and 4% were listed with the woman's last name and second initial, but not their first initial [6]. These prior findings identify a potential benefit in documenting how a female participant's telephone number is listed to facilitate locating them if they move. However, prospective evidence is not available. We examined predictors of locating older women last contacted between 1995 and 1997, who required tracing for recruitment into a new study completed between 2003 and 2004. We hypothesized that: 1) having the listed name for telephone numbers identified through a prior Internet search would facilitate locating women, 2) older women would be more difficult to locate, and 3) women residing in a high population density region would be more difficult to locate compared to those residing in a low population density region. The benefit of having access to vital statistics data (i.e., death records) was considered separately.

## Methods

### Study sample

We obtained contact information from a study completed of all persons aged 55 or more years residing within two regions of Ontario, Canada [7]. Of the 16,521 women who participated by completing a short screener questionnaire, 2,358 were eligible for a longitudinal study. The remaining 14,163 women were not contacted again and provided a list to sample from for our new study. Our primary estimates of interest were the prevalence of osteoporosis investigation and treatment within the two regions [8]: one densely populated within a metropolitan area (East York within Toronto; population density = 5,418/km^2^; land area = 21.26 km^2^), and the other largely a farming community (Oxford County; population density = 49/km^2^; land area = 2,039.44 km^2^) [9]. A total sample size of 1,500 was required to reach the objectives of our new study.

We searched a random sample of 750 women on the Internet in June 2001 to examine the feasibility of using the list (n = 14,163) as a sampling frame for our new study. These Internet search directories are based on the name, address and telephone number listed in public records, such as Canadian residential white pages [6]. This feasibility pilot study used web-search strategies to identify the listed name for telephone numbers and to estimate tracing resources required. Women were not contacted during this pilot study. Of the 750 women searched during the pilot study, 507 were eligible (e.g., community-dwelling, aged 65–89 years and alive based on a vital statistics linkage completed in August 2002) for the new study to begin recruitment in 2003. This sample of 507 women was supplemented with additional region-stratified random samples, for a total sample size of 1,500.

### Study recruitment and publicly available tracing strategies

#### Study web-search prior to recruitment (identifying listed name and address status)

We completed web-searches prior to recruitment (March to May 2003) for the 1,500 women sampled to identify telephone numbers for those without one at baseline, the listed name (as published in the directory) for each telephone number, and current address. Full names, complete address, and where available, telephone numbers from last contact (1995–1997) were printed on mailing labels and pasted on the web-search forms to blind those conducting the Internet searches to the results of the pilot web-searches completed in June 2001. Participants were first searched using 411.ca™ by telephone number or address using reverse lookup [10]. We then used forward search strategies by listed name (last name and first initial or first name) using two other websites [11,12]. These are public information websites that provide address, telephone number and name as listed in public records, such as residential white pages [6]. Based on these strategies, address status was coded as: 1) no change, 2) new address, 3) possible address – not enough information to have confidence in the address identified, or 4) unable to determine – could not find or too many possible listings to determine.

#### Study contact (mailings and telephone recruitment)

A newsletter introducing the study was mailed to the complete study sample (n = 1,500) in May 2003 using updated address information. If the address status was "possible" or "unable to determine," the newsletter was sent to the 1995–1997 contact address. Personalized information letters were then mailed in small batches depending on interviewer availability, timed to arrive a few days prior to study recruitment by telephone. Mailings were sent in envelopes with the study logo requesting that mail be returned if sent to the incorrect address. Mail returned to the study centre was used to identify women requiring tracing. These women were removed from the list of individuals for contact until they were located. Study recruitment began in May 2003 and lasted one year. All participants were offered her choice of a $10 gift card to a department store, bookstore or drug store.

#### Tracing

Women for whom a newsletter or information letter was returned, or for whom the telephone number was incorrect, required tracing. Within this group of women, the following strategies were used to identify their listed name: June 2001 pilot study web-search results (where applicable), searches of 1997 and 2000 telephone directory CD-ROMs, and if necessary and available, searches of city directories and telephone books [13-16]. Finally, a comprehensive web-search was completed to capture possible relatives, based on last name, who were then contacted by telephone [17]. This website was identified during recruitment to contain the most current address information. Tracing efforts ended in May 2004 coinciding with the scheduled end of study recruitment. Vital statistics linkages were completed in March 2004 and May 2005 to identify those who had died. Ethical approval was received from our institutional review board.

### Vital statistics linkages

We submitted a Data Request Summary to the Privacy Review Committee, Office of the Registrar General in June 2001. Permission was granted in May 2002 and vital statistics linkages were completed at Cancer Care Ontario in August 2002, March 2004 and May 2005. These data allowed us to determine if individuals had died.

### Statistical analysis

We tabulated study outset web-search results and the proportion of May 2003 newsletters that were returned, stratified by population density of the region. Regional differences were compared using the Pearson chi-square statistic. Logistic regression was used to determine the crude and adjusted odds ratio (OR) estimates associated with locating women requiring tracing. The following predictors were considered: age, study address status, whether or not web-searches were completed in 2001, population density, and the reason for tracing. We examined the final study status (participant, ineligible, refusal or unable to contact for participation) of those located using publicly available methods to gain a better understanding of the eligibility of those requiring tracing, and whether or not participation rates differed between eligible women located through tracing, from those who did not require tracing. Finally, among women not located using publicly available methods, we summarized the number and proportion identified as deceased through vital statistics linkages.

## Results

### Publicly available tracing strategies

#### Study web-search prior to recruitment (identifying listed name and address status)

Just over two-thirds (n = 1,030) of the 1,500 women sampled were identified at their 1995–1997 contact address (Table 1). Results were significantly different between the two regions (p < 0.0001). A new address was identified for 7% (n = 106) of women sampled, and of these 106, 82% (n = 87) resided in the less densely populated region. The remaining 364 women sampled were not located (9% possible addresses identified, 15% unable to determine) and thus the newsletter was mailed to their address at last contact (1995–1997).

**Table 1:** Study web-search results prior to recruitment (March–May 2003) using contact data from 1995–1997

	Population density of the region					
			
Study address status*	Low (n = 750)		High (n = 750)		Full Sample (n = 1,500)	
	n	(%)	n	(%)	n	(%)
No change	501	(66.8)	529	(70.5)	1030	(68.7)
New address	87	(11.6)	19	(2.5)	106	(7.1)
Possible address	53	(7.1)	82	(10.9)	135	(9.0)
Unable to determine	109	(14.5)	120	(16.0)	229	(15.3)

*Address status as determined by March–May 2003 web-search [10-12].

Low population density = Oxford County, population density = 49/km^2^; high population

density = East York within Toronto, population density = 5,418/km^2 ^[9].

#### Newsletter mailing

One fifth (n = 279) of the 1,500 newsletters mailed in May 2003 were returned to the study centre due to incorrect or incomplete address (Table 2). Overall, there were no differences between the proportion of newsletters returned by population density (17.7% low density vs. 19.5% high density, p = 0.39). The majority of the 279 newsletters returned were from women not located during the web-search completed prior to recruitment from March to May 2003 (n = 219, 78%). Of 106 new addresses identified in the study web-search prior to recruitment, 16 (15.1%) were returned. The return rate was higher among those sent to a new address, compared to those identified with no address change among women residing in the high population density region (47% vs. 4%, p < .0001), but not among those residing in the low population density region (8% vs. 4%, p = 0.12).

**Table 2:** Returned newsletters, overall and by study address status

	Population density of the region					
Returned newsletter*	Low (n = 750)		High (n = 750)		Full Sample (n = 1,500)	
Overall (n = 1,500)	133	(17.7)	146	(19.5)	279	(18.6)
**Study address status**^†^						
No change (n = 1,030)	21	(4.2)	23	(4.3)	44	(4.3)
New address (n = 106)	7	(8.0)	9	(47.4)	16	(15.1)
Not identified^‡ ^(n = 364)	105	(64.8)	114	(56.4)	219	(60.2)

Values expressed as number (percent).

*Newsletter mailed in May 2003 returned due to incorrect or incomplete address.

^†^Address status as determined by March–May 2003 web-search [10-12].

^‡ ^"Possible" address and "unable to determine" grouped since sent to last known address (results statistically similar: 59% of possible address and 61% of unable to determine were returned, chi-square = 0.24, df = 1, p = 0.62).

Low population density = Oxford County, population density = 49/km^2^; high population

density = East York within Toronto, population density = 5,418/km^2 ^[9].

#### Tracing

**Figure 1:**
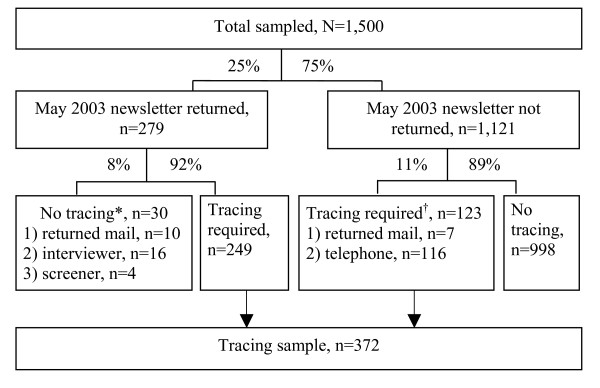
**Flow diagram of how sampled women were identified for tracing**. *Returned mail, but not traced due to: 1) mail returned included information to categorize the woman as ineligible, e.g., deceased; 2) contact by telephone interviewer before the mail was returned to the study centre; 3) 1995–1997 screener questionnaire included information to categorize the sampled woman as ineligible, e.g., plans to move out of the eligible regions. ^†^Identified as requiring tracing due to: 1) information letter returned in the mail to study centre or 2) incorrect telephone number at study contact by the telephone interviewer.

One quarter of the 1,500 women sampled (n = 372) required tracing. About a third (n = 116) of the 372 women who required tracing were identified as such by the telephone interviewer, at the time of study recruitment, when their telephone number was found to be incorrect (Figure 1). Table 3 summarizes characteristics of the women sampled and the proportions for whom tracing was required. Women requiring tracing were significantly older (mean = 77.0 years, SD = 6.7) than those who did not (mean = 75.4 years, SD = 6.4, p < 0.001). Fewer women from the less densely populated region required tracing (22% vs. 28%, p = 0.02). As expected, whether or not the woman sampled was part of the web-search pilot study was not associated with requiring tracing (25%; the study web-search prior to recruitment in 2003 was completed blind of the June 2001 pilot web-search results). Other than study address status (Table 1), there were no regional differences among the characteristics of women who required tracing.

**Table 3:** Characteristics of sample and proportion requiring tracing, n = 1,500

	Total sample		% Required tracing*
	n	(%)	(%)
Age group (years)			
65–69	313	(20.9)	(21.4)
70–79	721	(48.1)	(21.9)
80–89	466	(31.1)	(31.5)
Region (population density)^†^			
Low	750	(50.0)	(22.1)
High	750	(50.0)	(27.5)
Web-search in 2001			
Yes	507	(33.8)	(24.5)
No	993	(66.2)	(25.0)
Study address status^‡^			
No change	1030	(68.7)	(6.6)
New address	106	(7.1)	(16.0)
Possible address	135	(9.0)	(80.0)
Unable to determine^§^	229	(15.3)	(78.2)

*Proportion within each sub-category that required tracing.

^†^Low population density = Oxford County, population density = 49/km^2^; high population

density = East York within Toronto, population density = 5,418/km^2 ^[9].

^‡^Address status as determined by March–May 2003 web-search [10-12]; completed blind of 2001 web-search results.

^§^Not found or too many possible.

Forty-nine percent of the 372 women who required tracing were located using publicly available methods (Table 4). Focusing on adjusted estimates, women in their 80s had lower odds of being located compared with women in their 70s (OR, 2.13; 95% confidence interval (CI), 1.31–3.46). Although the difference between locating women in their 80s (39%) compared to women aged 65–69 years (55%) was substantial, it was not statistically significant after adjusting for other factors (OR = 1.73; 95% CI, 0.93–3.22). Fewer women residing in the densely populated region were located (OR = 0.55; 95% CI, 0.30–5.86). Whereas being unable to identify participants using the web-search prior to study recruitment decreased the likelihood of locating women, having had a web-search completed in 2001 was an independent facilitator.

**Table 4:** Proportion and predictors of locating women who required tracing, n = 372

	Located		Crude odds ratio		Adjusted* odds ratio	
	(n = 181)		OR	(95% CI)	OR	(95% CI)
	n	(%)				
Age group (years)						
65–69	37	(55.2)	1.95	(1.09–3.50)	1.73	(0.93–3.22)
70–79	87	(55.1)	1.94	(1.23–3.05)	2.13	(1.31–3.46)
80–89	57	(38.8)	1.00	(referent)	1.00	(referent)
Region (population density)^†^						
Low	91	(54.8)	1.00	(referent)	1.00	(referent)
High	90	(43.7)	0.64	(0.42–0.97)	0.55	(0.35–0.86)
2001 web-search^‡^						
Yes	70	(56.5)	1.60	(1.04–2.47)	1.82	(1.14–2.90)
No	111	(44.8)	1.00	(referent)	1.00	(referent)
Study address status^§^						
No change	41	(60.3)	1.00	(referent)	1.00	(referent)
New address	10	(58.8)	0.64	(0.25–1.69)	1.00	(0.33–3.02)
Possible address	66	(61.1)	0.93	(0.49–1.77)	1.09	(0.57–2.07)
Unable to determine	64	(35.8)	0.33	(0.18–0.60)	0.34	(0.19–0.61)
Reason required tracing						
Returned mail	128	(50.0)	1.00	(referent)		
Incorrect number	53	(45.7)	1.19	(0.77–1.85)		

CI = confidence interval.

OR = odds ratio.

*Adjusted for all variables indicated in the table (all variable except reason in tracing which was not significant).

^†^Low population density = Oxford County, population density = 49/km^2^; high population density = East York within Toronto, population density = 5,418/km^2 ^[9].

^‡^Included in pilot study having web-search completed in June 2001; results only used if tracing was required after the study web-search prior to recruitment (March–May 2003).

^§^Address status as determined by March–May 2003 web-search [10-12]; completed blind of 2001 web-search results.

#### Study status and participation rate

**Figure 2:**
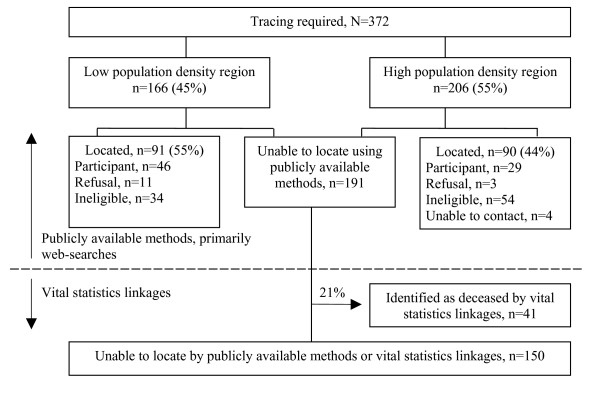
**Flow diagram and status results for sampled women requiring tracing**. Low population density = Oxford County, population density = 49/km^2^; high population density = East York within Toronto, population density = 5,418/km^2 ^[9].

Almost half of the 181 women located were ineligible for the study, primarily because they had moved out of the eligible regions (n = 54, 61%), or had died (n = 17, 19%), Figure 2. The participation rate among eligible women located was 84% (75/89).

### Vital statistics linkages

Of the 191 women not located by the end of participant recruitment (May 2004), 41 (21%) were subsequently identified as deceased through vital statistics linkages completed in March 2004 or May 2005, leaving 150 (10%) of the 1,500 women sampled for the study untraceable (Figure 2). However, the May 2005 linkage was only complete for deaths occurring prior to January 2003. It was estimated that vital statistics data at Cancer Care Ontario included most of the deaths occurring in 2003 (particularly for deaths occurring before August 2003), but that only 7% of the deaths occurring in the year 2004 were captured.

## Discussion

Our findings support the benefit of documenting the web-listed name for female participants' telephone numbers during recruitment to facilitate longitudinal follow-up. We endeavored to recruit women into a new study after six to eight years without contact. Although 25% of women sampled required tracing subsequent to a web-search completed in 2003, half were located by supplementing this search with: 1) the listed name for telephone numbers at last contact identified by a prior web-search (2001 feasibility pilot web-search), and using old telephone CD-ROMs, and 2) by broadening web-searches to capture relatives. Although this study is limited by not documenting the exact strategy that resulted in locating each woman, the finding that web-tracing completed two years prior to study recruitment was independently associated with locating women, provides compelling evidence of the benefit for researchers of longitudinal studies to include the listed name for telephone numbers identified through web-resources as part of their study design. Participation among eligible women located by tracing efforts in this study was 84%, comparable to the participation rate of 84% among those who did not require tracing [8,18]. This confirms prior reports that the main challenge in recruiting women who have moved is in locating them [2], and thus supports the importance of tracing to maximize response rates. As reported by others, however, caution is warranted in tracing study subjects to ensure that the person found is the person being traced [19]. In our study, four of the 'traced' women recruited were subsequently identified as the wrong person. Fortunately, we asked participants for their date of birth, which permitted us to identify these mistaken identity errors.

The Internet resources used in our study are limited by including only those residents listed in publicly available directories, such as residential white pages. Part of our inability to locate participants who required tracing may thus be related to them having unlisted telephone numbers. To overcome this limitation, we searched East York city directories housed at the Toronto reference library that include all residents, searchable by name, telephone number and address. Using reference library resources, however, did not appear to help identify hard to locate women from the metropolitan area. Nevertheless, city directories have not been printed in Canada since 2001, and may thus only be useful for searching for the listed names associated with an old address or telephone number [20]. However, such resources may be useful in areas that continue to publish city directories.

We documented the benefit of web-resources to locate women residing in a Canadian province. Similar resources based on residential white pages are available for numerous developed nations. For example, InfoSpace^® ^includes links to worldwide directories [11]. The existence of these resources suggests a potential opportunity to use similar strategies to those used in this study to trace participants. We also found that gender norms, regional differences and age influenced the success of tracing efforts. Prior research in Canada has found that female participants were primarily located under a male's name or the woman's second name or initial, but not their first name or initial [6]. We believe that for the same reason, having the listed name documented by a previous web-search facilitated locating women in our study. However, this finding needs to be examined further to assess its generalizability in other settings.

As people age, they may become more difficult to locate. Not only are older individuals more likely to die, they may move to live with other family members or reside in long-term care facilities. Expanding our web-search to capture potential relatives may have facilitated finding some 'hard to locate' women who moved to long-term care or to reside with relatives, as well as those with unlisted telephone numbers. However, we also found that tracing participants using web-resources was more difficult in the densely populated region due to a higher frequency of possible matched, particularly when expanding the search to capture possible relatives. It may thus be particularly prudent to document the listed name for participants residing in densely populated regions.

Finally, although vital statistics linkages were helpful to identify deceased individuals, access to these resources may be limited by privacy legislation, and the data may not be available in a timely fashion [21]. We found that vital statistics data were incomplete a year after the end of study recruitment. Vital statistics linkages may thus be better suited for post hoc adjustment of response rates than for on-going status identification among those who are hard to reach during study recruitment. However, when length of time since last contact is long, e.g., more than 5 years, an initial vital statistics linkage may help to exclude deceased prior to initial recruitment into the new study.

## Conclusion

Prospective studies may benefit from using Internet resources concurrent with baseline recruitment to determine the listed name for participants' telephone numbers. This information may then facilitate follow-up by helping to locate more women who require tracing, and thus also improve response rates. Although vital statistics linkages may help to identify deceased individuals, these data may not be accessible or current, and may thus be best suited for post hoc response rate adjustment.

## Competing interests

The author(s) declare that they have no competing interests.

## Authors' contributions

SMC contributed to study conception, design, and data collection, completed statistical analyses, and prepared the manuscript for publication. LD contributed with study coordination, data collection and manuscript review. MAMG, DEB, SBJ and GAH contributed to study conception, design and manuscript revision. All authors read and approved the final manuscript.

## Pre-publication history

The pre-publication history for this paper can be accessed here:


